# Modeling the Substitution of One Egg Increased the Nutrient Quality of Choline and Vitamin D in Exemplary Menus

**DOI:** 10.3390/nu17071129

**Published:** 2025-03-24

**Authors:** Analí Morales-Juárez, Alexandra E. Cowan-Pyle, Regan L. Bailey, Heather A. Eicher-Miller

**Affiliations:** 1 Department of Nutrition Science, Purdue University, West Lafayette, IN 47907, USA; moral146@purdue.edu; 2 Institute for Advancing Health Through Agriculture, Texas A&M AgriLife Research, Texas A&M University System, College Station, TX 77845, USA; alexandra.cowan@ag.tamu.edu (A.E.C.-P.); regan.bailey@ag.tamu.edu (R.L.B.)

**Keywords:** eggs, adolescents, modeling food substitution, Food Nutrient Index, exemplary menus

## Abstract

**Background/Objectives**: Eggs, a nutritious and affordable food, are not widely consumed by adolescents, who show many nutrient inadequacies. Modeling dietary substitutions with eggs and their costs can provide dietary insights while considering economic constraints. This study theoretically modeled the impact of substituting an egg for another protein source, considering nutrient quality and cost, using exemplary menus with application to adolescents. **Methods**: The substitution was modeled in four different seven-day exemplary menus: (1) the Healthy U.S.-Style Dietary Pattern (HUSS), (2) Harvard Medical School’s Heathy Eating Guide, (3) the National Heart, Lung, and Blood Institute’s Dietary Approaches to Stop Hypertension (DASH) diet and (4) the Healthy U.S.-Style Vegetarian Dietary Pattern (HVEG). One egg replaced the gram amount and nutrient profile of a protein source food in each menu. Micronutrient quality was assessed using the Food Nutrient Index (FNI), scored 0–100. The Center for Nutrition Policy and Promotion Food Price Database informed the food prices. Pairwise *t*-tests compared the effects of egg substitution on micronutrient scores and daily costs. **Results**: The daily egg substitution increased FNI scores for choline and vitamin D in the HUSS (83 to 95 and 69 to 75, respectively), DASH (80 to 91 and 55 to 59, respectively), and HVEG (91 to 100 and 44 to 51, respectively), and choline alone (89 to 98) in the Harvard menu. Daily menu prices were not significantly different after the egg substitution (*p* > 0.01). **Conclusions**: Substituting one egg for another protein source food increased the micronutrient quality of choline and vitamin D in exemplary menus without increasing the cost; however, factors such as food preferences and the economic accessibility of eggs in different contexts should also be considered.

## 1. Introduction

Adolescents are in a rapid and final stage of growth and development that will set the trajectory for their adult health [1,2]. Certain nutrients, such as protein, the B vitamins, vitamins C and D, choline, calcium, magnesium, potassium, selenium, iron, zinc, iodine, and omega-3 fatty acids such as docosahexaenoic acid and α-linolenic acid, are critical to healthy growth during this time, especially bone and brain health [3,4,5,6,7]. For example, bone formation in adolescence depends on protein, vitamins C and D, calcium, magnesium, potassium, and others to accumulate peak bone mineral content [3,4,5], while brain development and executive function (e.g., neurocognitive process such as attention and focus, behavior, emotions, memory, and learning) depend on nutrients including folate and vitamin B12, choline, iron, zinc, iodine, and omega-3 polyunsaturated fats [6,7]. However, the quality of dietary intake during adolescence is at the lowest level seen across the entire lifespan. This life stage is also when the most nutrient gaps in meeting adequacy markers are experienced [2]. Nearly all adolescents fail to meet vitamin D (~90% < the estimated average requirement) [2,8] and choline (only ~15% > the Adequate Intake) [8,9] adequacy, which is similar to other age groups [2,9]. However, poor adequacy for protein, folate, and iron (mainly among females) and vitamin B6, vitamin B12, phosphorus, and magnesium (for both females and males) constitutes a unique constellation of nutrient risk in this life stage compared with others [10,11]. Eggs are a source of these nutrients and more, including vitamins A, B6, B12, choline, vitamin D, vitamin E, folate, riboflavin, thiamin; calcium, iron, potassium, selenium, and zinc; lutein and zeaxanthin; and omega-3 fatty acids including docosahexaenoic acid and α-linolenic acid [12,13]. The bioavailability (e.g., the proportion of a nutrient in a food that is absorbed and used for normal body function) [14] of many nutrients in eggs is also high [15,16]. For example, the bioavailability of folate is approximately 72% from cooked eggs, 50% from cooked beef liver, 69% from chickpeas, and 37% from tomatoes [15]. For vitamin B12, the bioavailability is about 76% from soft boiled eggs, 63% from chicken meat, 55% from enriched white bread, and 65% from enriched milk [15]. Choline from egg yolks and whole eggs have bioavailability of 101% and 104%, respectively, compared to supplementary choline [15], and lutein within enriched eggs is also more bioavailable than that from supplements or spinach [16].

Eggs are also low-cost and widely consumed in the population and across several population groups by race, ethnicity, culture, income, sex, and age group [17]. In adolescents, eggs and omelets ranked among the top 25 most frequently consumed foods (12–17 years) [18]. Eggs can be incorporated into a menu in many ways, as the main food in several dishes (e.g., omelet, boiled egg) and also as ingredients (e.g., egg burrito, stir-fried rice, crepe). Therefore, eggs offer a strategy for improving the intake of protein, vitamin D, choline, and many others that are important for the growth and development of adolescents.

Evidence-based exemplary menus have previously been developed to practically outline specific foods that could be included in a daily menu to fulfill nutrient intake goals for diets at various kilocalorie levels [2,19]. These menus can be selected to fit certain health or dietary preference or other needs. The Healthy U.S.-Style Dietary Pattern (HUSS) [20] aims to promote health and prevent disease in alignment with the U.S. Dietary Guidelines for Americans (DGA). The National Heart, Lung, and Blood Institute’s Dietary Approaches to Stop Hypertension (DASH) diet menu [21] is aimed at lowering blood pressure. The Harvard Medical School’s Heathy Eating Guide (Harvard menu) promotes overall health but includes less dairy in the pattern [22], and a vegetarian style of dietary pattern can be implemented using the Healthy and Vegetarian U.S.-Style (HVEG) Dietary Pattern menus [23]. Exemplary menus offer practical guidance to meal-based programs and dietary interventions to promote health (for example, the National School Lunch Program and the National School Breakfast Program [24]). The incorporation of eggs into exemplary menus and a consideration of their estimated cost differential could provide insights into the nutrient quality provided through the implementation of such menus in programs and future interventions directed to adolescents. In 2020, it was estimated that the cost of eggs was approximately USD 0.35 per 100 g [25] and they have been ranked as the most cost-efficient food for delivering protein, vitamin A and choline; second for vitamin E; and third for vitamin D among those 2–18 years of age using the mean cost per unit (g, mg, or mcg) of nutrients compared to other protein food subgroups, as defined by the U.S. Department of Agriculture (USDA) [25]. When combined with other nutrient-dense sources, eggs can be part of a high-quality diet, implemented through exemplary menus that may be used to address nutrient gaps [6].

Eggs have not been frequently incorporated into exemplary menus, but their affordability and nutrient composition may offer a way to improve nutrient quality while not increasing the cost. Nutrient quality can be estimated using the Food Nutrient Index (FNI) [26], a scoring system (from 0 to 100) that evaluates micronutrient intakes from foods and beverages relative to the Recommended Dietary Allowance or Adequate Intake for underconsumed micronutrients [26,27]. Using the FNI, the nutrient quality of exemplary menus can be compared in the context of modeling, by strategically substituting an egg for a different protein source food in the existing menu; price analysis can be utilized to compare the prices of resulting menus with and without substitution. Therefore, the objective of this study was to theoretically model the impact of substituting one medium egg (44 g) for another protein source food while holding energy constant on the nutrient quality (via the FNI) and average daily price (USD) of four exemplary menus of relevance among U.S. adolescents (14–18 years) at their age-appropriate kilocalorie levels.

## 2. Materials and Methods

### 2.1. The Exemplary Menus

Very few exemplary menus to promote overall health and lower disease risk are available, and there are none specifically for adolescents. However, four different menus fulfilling these goals were evaluated in this cross-sectional modeling analysis, to determine the impact of egg substitution on FNI scores and cost estimations: (1) the seven-day 2000 kcal/d HUSS [20]; (2) the seven-day 2000 kcal/d DASH diet menu [21]; (3) the seven-day 2000 kcal/d Harvard menu [22]; and (4) the seven-day 2400 kcal/d HVEG [23]. These exemplary menus varied in total energy intake from 2000 to 2400 kcal, which correspond with the estimated energy requirement [28] for adolescent females (14–18 y; range: 1800 to 2400 kcal/d [2]), and which are slightly lower than those for adolescent males (14–18 y: range: 2000 to 3200 kcal/d [2]). For this reason, the estimated total energy intake for each menu plan was not altered for the present analysis. The HUSS menu was created through the food-based modeling of the DGA recommendations while meeting energy needs and fulfilling nutrient requirements for each sex–age group in the U.S. [20]. The DASH diet menu is centered around a dietary pattern aimed at lowering blood pressure, and thus includes dietary components designed to yield higher potassium, magnesium, and calcium intakes, and lower sodium intake relative to a common U.S. eating pattern [29]; this is achieved through the increased consumption of fruits, vegetables, whole grains, lean meats, and low-fat dairy. The Harvard menu includes a set of evidence-based nutritional recommendations focused on promoting long-term health and preventing chronic diseases through a balanced and sustainable approach to diet [22]. The Harvard menu shares similarities with the HUSS and the DASH diets in its emphasis on fruits, vegetables, whole grains, and lean meats [20,21,22]. However, the Harvard menu limits the intake of animal-derived milk or other dairy products to one to two servings/day [22]. The HVEG is a variation of the HUSS that includes many of the same core elements (i.e., vegetables, fruits, grains, dairy, protein foods and oils) to meet nutrient requirements, but is not inclusive of animal flesh/products and instead comprises eggs, beans, peas, lentils, soy products, nuts, and seeds [23].

All four exemplary menus include nutrients from food and beverages only, and not from dietary supplements. The nutrients provided by each menu were calculated by staff at the Purdue University Diet Assessment Center using the Nutrition Data System for Research software versions 2021 and 2022 developed by the Nutrition Coordinating Center, University of Minnesota, Minneapolis, MN [30]. Review by the institutional review board was not required for this study because human subjects were not involved, as per U.S. Department of Health and Human Services guidelines [31].

### 2.2. Modeling Egg Substitution

For each of the four exemplary menu examples, the main protein source foods were identified across a seven-day menu cycle (Supplementary Materials). The protein source foods were identified based on the DGA’s designation of foods in the protein food group, which includes meats, poultry, and eggs; seafood; beans, peas, and lentils; and nuts, seeds, and soy products [2]. The protein content (in grams) was then compared across these protein source foods within each daily menu to identify the protein source foods with the highest protein content, which was selected as the protein source food for substitution. The protein source foods selected for substitution were also chosen with consideration of dietary variety and to maintain adherence to DGA recommendations. For example, the HUSS menu included “Salmon, cooked from fresh or frozen” and “Roast beef” as the main protein source foods; “Roast beef” was selected for the egg substitution, while salmon remained within the menu to meet the DGA’s suggestion of eight-ounce equivalents per week from seafood in a 2000 kcal menu. Similarly, varying protein over all of the days was prioritized when making the egg substitution. For example, in the same menu plan, if “Steak-beef” and “Lunchmeats and sausages, chicken” were the main protein source foods on a given day within the seven-day menu plan, the protein source food selected for substitution was “Lunchmeats and sausages, chicken” given that a “Steak-beef” protein source option was already selected for the egg substitution (i.e., the “Roast beef”) on a different day within the menu plan. Since 44 g of one egg provides a lower gram amount of a protein source food than the exemplary-menu-prescribed amount of each protein source food selected for the substitution (e.g., 168 g of salmon), the entire protein source was not fully replaced. As a result, only partial substitutions were implemented, and the remaining portion of the original protein source was retained in the menus. For example, instead of replacing all the salmon with around four eggs, only a portion of the protein source food in the menu was substituted with one egg (e.g., 44 g of the other protein source). This partial substitution makes the approach more realistic and practical because it maintains the nutritional balance of the menu by preserving the amount of the protein source food while incorporating a wider variety of foods, making meal plans easier to follow and adhering to the DGA goal of variety.

Most of the protein sources were single-item foods (e.g., “Steak-beef”), and there were only four mixed dishes (e.g., spaghetti with tomato sauce and meat) (Supplementary Materials). The balance of flavors, textures, and nutrients was considered for this theoretical modeling of an egg substitution, especially for the mixed dishes, where protein sources were combined with other ingredients and the substitution needed to complement the existing flavors and textures without overpowering the dish. By using partial substitutions in both single-item foods and mixed dishes, the study provides a more accurate representation of how individuals might realistically incorporate eggs or other substitutes into their meals without compromising nutrient intake or the overall appeal of the meals. For instance, for foods in simple preparation forms, instead of serving only turkey as a protein source, a boiled egg can be included, with the rest of the protein source food intake fulfilled by turkey. This partial substitution retains the nutritional content of turkey while adding the unique nutrients found in eggs. Similarly, in a mixed dish such as a chicken salad, one boiled egg can be added by slightly reducing the amount of chicken. This approach provides variety and enhances the meal’s nutrient profile without sacrificing protein intake or taste.

Next, in each menu, the gram amount of one medium egg (44 g) was substituted for the same gram amount of the protein source food in the menu chosen for the substitution, using an “Egg, boiled (hard or soft)” with food code 975 also from the Nutrition Data System for Research. A boiled egg was selected because of its simple preparation process, without the need to add any other ingredients such as seasonings or dietary components. Accordingly, all of the nutrients comprising the selected protein source food from the menu were removed and the nutrients comprising one medium egg were added.

### 2.3. Estimation of Mean Daily Menu Prices

The Center for Nutrition Policy and Promotion Food Prices Database [32,33] from the USDA was used to estimate the mean daily price of each menu. The latest iteration of the Center for Nutrition Policy and Promotion Food Prices Database was utilized because it contains the average national prices of approximately 4600 food items in their “as-consumed” state. This differs from “as-purchased” data, as it considers alterations in weight due to cooking and excludes waste, such as vegetable peels and meat bones [32]. The Center for Nutrition Policy and Promotion Food Prices Database contains the price (USD) per 100 g of each individual food, so the price was calculated for the amount of each food in the menu based on this conversion. Nine spices (e.g., paprika) used in the HVEG menu were not found in the Center for Nutrition Policy and Promotion Food Prices Database and were not included in the price estimation; however, the amount of spices included in the recipes was relatively small (between 1 and 2 g). When a food item from the exemplary menu was not found in the Center for Nutrition Policy and Promotion Food Prices Database, the most similar one was used; this occurred only with nine food items from the Harvard menu (e.g., olive swirl rolls, lemon cilantro aioli, and onion-crusted tofu). Finally, the menu price was estimated per day and a mean daily price and standard deviation were obtained using seven days per exemplary menu.

### 2.4. Statistical Analyses

Eight nutrients that are underconsumed among adolescents were considered in the daily total and mean menu nutrient models used to calculate the FNI score [2,8,9,10,11,26]. Vitamins C and D, calcium, magnesium, and potassium are essential for bone health as bone growth reaches its peak before the growth plates close [3,4,5]. Folate, choline, and zinc play important roles in brain development, encompassing cognitive, emotional, and neurological changes [6,7]. Therefore, the daily totals of these eight underconsumed micronutrients were summed for each menu, then the mean daily amounts of each micronutrient were estimated across all seven menu days for each menu as given and with the egg substitution. The FNI scores served as indicators of nutrient quality and were determined using the National Cancer Institute’s simple algorithm method for each of the four exemplary menus, both as given and with the substitution of one egg per day for the similar gram amount of another protein source food while holding energy constant. The FNI is a nutrient-based scoring system designed to assess nutrient quality from foods and beverages relative to the Dietary Reference Intakes for micronutrients that are underconsumed and are recognized by the DGA as being of public health concern [26,27].

For the FNI estimation, the mean of the eight nutrient values modeled across the seven days of each menu were used to calculate the percentage of Recommended Dietary Allowances or Adequate Intake (as applicable), specific for age and sex among adolescents 14–18 y [26]. The maximum score that could be assigned to a FNI component was 100. For example, if the amount of calcium in the exemplary menus exceeded the Recommended Dietary Allowance (1300 mg/d) for adolescents, the score assigned would be 100 [26]. If no calcium was present in the menu, a score of zero would be assigned. However, if the calcium amount in the menu was lower than the Recommended Dietary Allowance (e.g., 624 mg/d), the score was determined as a percentage of the Recommended Dietary Allowance based on the amount present in the menu (e.g., FNI for calcium = 48, because 624 mg/d from calcium represents 48% of the Recommended Dietary Allowance = 1300 mg/d) [26]. These percentages were determined for each FNI component score, where higher scores indicate closer alignment with the Recommended Dietary Allowance or Adequate Intake [26]. The overall FNI score was then calculated as the average of the component scores, with each component given equal weight [26,27].

The FNI prioritizes the inclusion of underconsumed nutrients based on scientific evidence of their health impact; however, it focuses solely on micronutrients and does not consider the macronutrient balance or overall diet quality [26]. Other nutrient profiling tools, such as the Nutrient-Rich Foods 9.3 Index, which focuses on nine nutrients to encourage (e.g., calcium, fiber, iron) and three to limit (i.e., saturated fat, added sugars, sodium), assigns equal weight to both positive and negative nutrients due to a lack of consensus on the strength of nutrient–health relationships [34]. In contrast, there are other quality scores that use proprietary algorithms, such as the Weighted Nutrient Density Score, which assigns nutrient weightings based on the health impact of nutrient imbalances, considering complex nutrient interactions [35]. In comparison, the FNI focuses on a broader range of nutrients without relying on complex algorithms or proprietary weightings [26,27]. Additionally, FNI scores effectively differentiate groups with varying dietary quality and correlate well with the biomarkers of nutritional status, demonstrating relative validity [26,27]. The FNI also has the advantage of being easily compared to the Total Nutrient Index (TNI), which uses the same scoring system but includes dietary supplements, making it identical to the TNI for the measurements of nutrient amounts that exclude supplements [26,27].

We used pairwise t-tests with a Bonferroni-adjusted *p*-value to account for multiple comparisons based on the number of comparison groups across all of the nutrients analyzed to determine statistically significant differences in the mean nutrients modeled. This adjustment was conducted to control the risk of producing false positives when testing multiple hypotheses. For the total nutrients per day, eight nutrients were analyzed across eight days (including the daily mean values), resulting in 64 possible pairwise comparisons, calculated as 8 × 8 = 64. With four menus, this led to a total of 256 (64 × 4) comparisons that were utilized in the Bonferroni adjustment of 0.05/256 for the *p* value = 0.0001. For the FNI total scores, two groups were analyzed (menu as given and with the substitution), resulting in one possible pairwise comparison. With four exemplary menus, there were 4 (1 × 4) comparisons that were utilized in the Bonferroni adjustment of 0.05/4 for the *p* value = 0.01. Similarly, for price comparisons, two groups were analyzed (the menu as given and with the substitution), also resulting in one possible pairwise comparison per menu. With four exemplary menus, this again totaled 4 (1 × 4) comparisons that were utilized in the Bonferroni adjustment of 0.05/4 for the *p* value = 0.01. All statistical analyses were completed using SAS 9.4 software (SAS Institute, Cary, NC, USA).

## 3. Results

### 3.1. The Total Nutrients per Day and Mean Daily Nutrients Across the Exemplary Menus

The total nutrients per day and mean daily nutrients modeled across all menu days for each exemplary menu, as given and with the substitution of one egg, are shown in Table 1. Overall, the HUSS and the HVEG exemplary menus were most strongly aligned with nutrient standards of adequacy (i.e., the Recommended Dietary Allowances and/or Adequate Intake) for adolescents 14–18 years, when compared with the DASH and Harvard menus. For both the HUSS and the HVEG menus, the mean daily amounts of six out of the eight micronutrients modeled (i.e., folate, vitamin C, calcium, potassium, magnesium, and zinc) were higher than their respective Recommended Dietary Allowances or Adequate Intake. The DASH menu was also closely aligned with the Dietary Reference Intakes for the same six nutrients above. However, choline and vitamin D modeled daily from the DASH menu did not meet the Dietary Reference Intakes, unlike the HUSS menu and the HVEG menu. Finally, the amount of only four out of eight micronutrients in the menus (folate, vitamin C, potassium and magnesium) exceeded the Recommended Dietary Allowances or Adequate Intake in the Harvard menu (Table 1). Among the four exemplary menus, mean daily choline increased when modeled with an egg-substituted (HUSS menu: from 387 mg to 497 mg; DASH menu: from 369 mg to 453 mg; Harvard menu: from 425 mg to 532 mg; and HVEG menu: from 452 mg to 563 mg). Mean folate and calcium also increased with the egg substitution for the HUSS, DASH, and Harvard (folate only) menus. Despite these small nutrient increases with the egg substitution, none were statistically significant (*p* > 0.001). Without the egg substitution, the mean amounts of choline for the four exemplary menus were also not above the Adequate Intake (i.e., 475 mg) but, with the substitution of one egg, the mean values of choline exceeded the Adequate Intake for the HUSS (497 mg), Harvard (532 mg), and HVEG (563 mg) menus.

### 3.2. Mean FNI and Component Scores Across the Exemplary Menus

The mean FNI and component scores across all four exemplary menus, as given and with the substitution of an egg, are shown in Table 2. The HUSS, DASH, Harvard, and HVEG exemplary menus received very high total scores on the FNI, ranging from 87 to 96. The Harvard menu obtained the lowest total FNI score, whereas the HUSS menu had the highest score, when both were compared with all other exemplary menus. For folate, vitamin C, magnesium, and potassium, the four exemplary menus attained the highest possible scores. However, none of the menus achieved a perfect score for vitamin D or choline (FNI component scores ranging from 44 to 69 and from 80 to 91, respectively). For the Harvard menu, calcium (FNI component score: 48) and zinc (FNI component score: 94) also failed to score perfectly. The FNI scores for choline increased with the substitution of an egg for the HUSS menu (from 83 to 95) and the DASH menu (from 80 to 91). The Harvard menu (from 89 to 98) and the HVEG menu (from 91 to 100) also improved.

Furthermore, the substitution of one egg per day improved the FNI vitamin D scores in the HUSS (from 69 to 75), DASH (from 55 to 59), and HVEG (from 44 to 51) exemplary menus. Overall FNI scores showed no significant differences in nutrient quality between the exemplary menus with and without the substitution of an egg, given that many already were at the maximum score of 100 unless noted above. Increases in both choline and vitamin D were only numerically relevant, as no statistical test was conducted on the individual components of the FNI.

### 3.3. The Total Price per Day and Mean Daily Price Across the Exemplary Menus

The total price per day and mean daily price across all menu days for each exemplary menu, as given and with the substitution of one egg, are presented in Table 3. On average, the DASH menu had the lowest average daily price (USD 6.1), as opposed to the HUSS menu, which had the highest price (~USD 10). Among the four exemplary menus, no significant differences were observed when comparing the mean daily menu prices of the exemplary menus as given and with the egg substitution.

## 4. Discussion

Adolescence is the life stage with the poorest diet quality in the U.S. [2]. Exemplary menus operationalize a high-quality diet that may be enhanced by the inclusion of nutrient-dense, low-cost, and frequently consumed foods, which are easily incorporated into daily meals with the potential to improve nutrient quality [2,19]. The current study modeled mean daily nutrients and totals, FNI scores, and average daily menu prices with and without the substitution of one egg per day for another protein source food while holding energy constant in four exemplary menus with application to adolescent diets. As expected, all of the exemplary menus exhibited high nutrient quality; however, for choline and vitamin D, shortfalls persisted across the four menus. The resultant findings also showed that substituting one egg for other protein-rich foods increased the FNI component scores for choline, regardless of the exemplary menu. This supports the notion that substituting one egg for other protein source foods can play a role in alleviating the risk of choline inadequacy among U.S. adolescents, especially when used in combination with an existing high-quality diet. FNI scores for vitamin D modestly improved when modeled with the substitution of an egg for the HUSS, DASH, and HVEG menus, a nutrient of public health concern where any increase may be viewed as a success for this broadly underconsumed nutrient [2]. The average daily menu prices, with and without the substitution of one egg, were not significantly different. The current modeling analysis supports the improvement in nutrient quality that may be imparted by the substitution of eggs for other protein sources using exemplary menus, without an increase in cost.

Eggs are a primary contributor to choline in the U.S. diet [36]. Choline is a precursor for acetylcholine, a neurotransmitter that is vital for memory, learning and attention regulation [36,37]. Choline also supports the development of the brain’s structure and function through its role in phospholipid synthesis [37], a key component of cellular membranes, which are essential for maintaining membrane structure and function, as well as facilitating intracellular signal processes [38]. High choline intake during pregnancy and early development in rats and mice has been shown to improve memory, slow age-related decline, and protect the brain from conditions such as Alzheimer’s, epilepsy, fetal alcohol syndrome, and genetic disorders such as Down and Rett syndromes [39]. These benefits may result from changes in deoxyribonucleic acid (DNA) and histone methylation, which affect genes linked to learning and memory [39]. Although animal studies may not directly translate to humans, studies assessing the impact of choline intake during pregnancy on child cognition show that choline supplementation during critical developmental periods improves memory and learning performance later in life [6]. Another study showed maternal choline supplementation at twice the recommended intake during the third trimester during pregnancy improved infants’ information processing speeds [40]. These studies indicate the neurocognitive importance of choline and its potential importance for adolescents as their brains undergo significant structural and functional maturation [1]. By around age six, the brain reaches approximately 90% of its adult size; however, the grey and white matter components continue to experience changes during adolescence [1,41]. Neuroimaging studies have shown that brain structure continues to develop into early adulthood, while the executive and social brain networks undergo functional maturation during adolescence [41]. Although adolescence is recognized as a critical window for cognitive development due to the ongoing structural maturation of the brain, evidence exploring the role of choline in adolescent cognitive outcomes remains limited. One study found that plasma choline levels were significantly and positively associated with academic achievement (e.g., school grades), independent of socioeconomic factors such as paternal and maternal education and income, smoking status, and school, as well as folate intake (*p* = 0.009, R^2^ = 0.29), among Swedish adolescents [42]. The investigation described in this study serves to highlight that substituting one egg for other protein source foods in a daily diet could increase the modeled mean daily nutrients above the Adequate Intake for choline; thus, eggs have the potential to bridge nutrient gaps for choline, specifically among adolescents.

Eggs also supply vitamin D (0.957 mcg) [43,44], a dietary component of public health concern in the U.S. population. The daily substitution of one egg increased the FNI scores for vitamin D modestly for three of the four menus (HUSS, DASH, and the HVEG menus). Eggs are one of the few food sources rich in vitamin D [45,46]; however, in this study, the modest increases in the FNI scores for vitamin D may be attributed to the relatively low vitamin D content in standard eggs. To improve vitamin D content in exemplary menus, additional modifications could be considered. For instance, laying hen diets could be supplemented with higher levels of vitamin D_3_ and 25-hydroxyvitamin D_3_; this could produce eggs containing between 100 and 500 IU of vitamin D, meeting the daily requirements for adults and children without affecting egg production [47]. In addition, the inclusion of other foods that are naturally rich in vitamin D, such as salmon, and those fortified with vitamin D, such as certain dairy products and cereals, could significantly boost the amount of vitamin D in menus [48]. In childhood and adolescence, vitamin D plays a critical role in calcium metabolism and fosters bone growth and bone mineral density [47,48]. Adolescence is a window of opportunity for the maximum acquisition of bone density across the lifespan, after which bone deposition does not keep up with losses. Ultimately, this situation may result in a risk of fracture in adulthood if adolescent bone density does not start out sufficiently high or if losses are too great. Therefore, any improvement in vitamin D and calcium intake during adolescence may support future adult bone health and decrease fracture risk [48]. Beyond its impact on skeletal health, which involves maintaining regular bone functioning and mineralization in adulthood, vitamin D might also offer a defense against various health issues, including type 1 diabetes mellitus, hypertension, multiple sclerosis, and cancer [48].

Previous studies have modeled the impact of adding or removing eggs on adolescent diets using actual intake data instead of utilizing exemplary menus. For example, a prior study modeled the incorporation of eggs in the breakfast of the Child and Adult Care Food Program, a meal program operated at the U.S. federal level to benefit children and adults in childcare centers, daycare homes, and adult daycare centers [49]. The study showed an increased percentage of children above the Adequate Intake level for total choline from 22.6% at baseline to 43.6% with one egg and 57.8% with two eggs [50]. In that study, the usual intake of vitamin D and other nutrients (pantothenic acid, riboflavin, selenium) also increased ≥10 percent (relative to the baseline values) [50]. Another study showed that modeling the addition of seven eggs per week increased the percentage of adolescents with intakes above the choline Adequate Intake from 22.1% (zero eggs added) to 52.5% in children 2–18 years [51]. Adolescents who were food secure and consumed primarily egg-based dishes had significantly higher mean usual intakes (*p* < 0.0002) of lutein + zeaxanthin, choline, selenium, vitamin D, vitamin B2, DHA, and protein compared to those who consumed eggs less frequently (e.g., as ingredients in mixed dishes) or did not consume eggs and/or experienced food insecurity. Notably, modeling the addition of one egg per day improved the nutrient intakes in all groups, with modeled intake for food-insecure adolescents showing benefits. For instance, modeling the addition of one egg per day improved the proportion of food-insecure adolescents with Adequate Intake for choline from ~3–33% to 22–63% [8].

Guided by the evidence showing the benefits of including eggs in adolescents’ diets and the results of the modeling conducted in this study, the inclusion of eggs in daily meals has the potential to benefit adolescents’ health, a period in which nutritional risks can be exacerbated in the context of inadequate economic resources for food and food insecurity [52]. However, the practical application of the egg substitution may vary in real-world settings. Factors such as dietary preferences, cultural food practices, and socioeconomic constraints could influence the feasibility of incorporating eggs into adolescents’ diets [53]. Additionally, the impact on meal palatability should be considered, as changes in taste and texture may affect acceptance [53]. Future studies should directly test egg substitution to evaluate acceptability and adherence, especially among different populations, including those facing food insecurity or certain dietary restrictions, to determine whether nutrient deficiencies are mitigated. Long-term studies would also help to determine whether substitution leads to sustained improvements in nutrient intake, cognition, and overall health. Likewise, the future implementation of a food education program tailored to meet nutritional needs that incorporates egg substitution could be considered. This type of program has shown promising results in terms of enhancing adolescents’ knowledge about nutrition and fostering their self-regulation skills related to dietary habits (e.g., goal setting, planning, and resisting temptations) [54]. The implementation of these practical aspects could help to translate the study’s findings into actionable dietary recommendations. Moreover, the results of this study could be used to guide the implementation of egg-inclusive menus in public schools and in programs targeting low-resource audiences. Furthermore, the cost of eggs (USD 2.4 medium eggs per dozen, average price combined regional in 2024) [55] may help keep menus within budgets both for programs and households with low incomes, which may not have the ability to afford other options.

In the Center for Nutrition Policy and Promotion Food Prices Database, 2003–2004, the price of a dozen medium eggs (44 g per egg) was USD 1 (i.e., USD 0.2 per 100 g of egg). This is an important price difference, compared to the more current USD 6.49 medium egg per dozen [56], indicating the potential inflationary trend in food prices over time. The prices in the Center for Nutrition Policy and Promotion database reflect average national prices that may not capture regional variations or seasonal fluctuations, so these prices may not accurately reflect the current market conditions. This limitation impacts the analysis, as it did not consider the effect of inflation on food prices, potentially leading to inaccuracies in estimating the cost of a balanced diet. The USDA Economic Research Service reported that food-at-home prices increased by 3.3% in 2020 and 2021, which was higher than the average annual increase over the past 20 years [57]. This trend indicates that relying on older data, such as the Center for Nutrition Policy and Promotion database from 2003–2004, may lead to an underestimation of the current cost of food items, including eggs. More recently, egg prices have experienced greater volatility due to factors such as supply chain disruptions and disease outbreaks, further complicating cost estimates [57]. Future analyses could account for inflation-adjusted prices or incorporate real-time market data to provide a more accurate assessment of dietary costs and affordability.

Additionally, regional price variations in eggs could be considered, as inflation can disproportionately affect different areas, further influencing food accessibility and affordability. According to recent USDA data, egg prices vary by region. A report for 7 March 2025, showed that the national average price for medium eggs was USD 6.49 per dozen [56]. Regional variations included an average of approximately USD 6.20–USD 6.46 in the Midwest [56], USD 6.56 in the South-Central region [56], USD 6.54 in the Southeast, and USD 6.44 in the Northeast [56]. This price variation suggests that regional economic factors may influence food affordability and access, which can impact dietary choices, specifically among food-insecure populations. When compared to other protein sources, eggs can be relatively affordable; the national average price per dozen for eggs of USD 6.49 translates to a price per pound of USD 4.94 (e.g., the weight per dozen medium eggs is 21 ounces [58]). In contrast, the prices of other proteins such as beef brisket ranged from USD 1.69 to USD 6 per pound [59], and turkey breast prices fell between USD 2.75 and USD 2.95 per pound [60]. In contrast, pasture-raised pork averaged USD 10.56 per pound [61] and skirt steak was USD 15.99 per pound [59], representing more expensive options that may be less accessible to low-income households [62]. Considering these cost differences, eggs, particularly in regions with lower prices, may offer a cost-effective strategy for enhancing nutrient intake. Incorporating this variability into dietary cost assessments is essential for evaluating the economic feasibility of dietary interventions. Further research should explore regional price differences and their potential impact on dietary quality and food security strategies.

In this analysis, comparing daily menu prices between the original menus and those with egg substitutions over a seven-day period revealed no significant differences. However, when projecting these differences over the span of a year, variations emerged. For example, for the HUSS menu, the average price over seven days is USD 9.6, while, with the egg substitution, it is USD 9.5. Extrapolating this information over a year results in respective totals of USD 3533 and USD 3492, showcasing a decrease in the menu price of USD 41 due to the egg substitution. Although this decrease in the menu price shows a relatively minimal difference, this price difference could be important for programs where these costs are multiplied by the number of people receiving the program. Moreover, the egg substitution in this analysis improved the nutrient profiles of choline and vitamin D without incurring extra costs. The Thrifty Food Plan, a nutritious and lowest-cost USDA food plan which includes the HUSS menu, had an average weekly cost of USD 64 for adolescents aged 14–19 years in April 2024 [63]. Similarly, the USDA Food Plans, offering nutritious diet at three different cost levels, reported average weekly costs of USD 65 for the low-cost plan, USD 79.15 for a moderate-cost plan, and USD 94.7 for a liberal plan aimed at adolescents aged 14–18 years [64]. In contrast, the current analysis of exemplary menus incorporating egg substitutions showed a lower mean weekly cost of USD 50 compared to both USDA food plans. Therefore, integrating egg substitutions into the Thrifty Food Plan and USDA Food Plans can be deemed a cost-effective strategy to reduce overall food plan expenses.

One advantage of modeling egg substitution in exemplary menus is that it is a way to evaluate an eating plan at a given energy level to determine what changes may occur without actually carrying out a more time-intensive and expensive feeding trial [19]; this means that a food substitution, e.g., of eggs, can be conducted theoretically. Although the HUSS, DASH and Harvard menu plans were created to meet 2000 kcal/day for seven days and the HVEG meal plan to meet 2400 kcal for seven days, slight discrepancies were noted in a few days of the plans (ranging from 1749 to 2244 kcal/day for the HUSS, DASH and Harvard menus and from 2400 to 2579 kcal/day for the HVEG menu). These variations in energy modeled might contribute in part to the differences observed in the FNI scores. To conduct an energy level standardization of the menus, some strategies could be implemented. First, the adjustment of the portion sizes and selection of foods with consistent energy densities could help to minimize discrepancies. Second, establishing clear criteria for acceptable calorie ranges within each exemplary menu could help to manage unavoidable variations. Finally, sensitivity analyses that account for energy differences could provide insights into the extent to which caloric variation impacts micronutrient quality scores.

Several strengths of this study can be noted. This is the first evaluation of exemplary menus following the substitution of one egg for other protein food sources, focused on underconsumed nutrients among adolescents. Food substitution analysis is valuable for determining the ideal food composition in a diet, making it instrumental for informing decisions among public health policymakers [65]. This modeling of an egg substitution was conducted while addressing various eating preferences and patterns by evaluating a range of exemplary menus, such as those recommended for a low-sodium diet, a diet lower in dairy products, a general healthy diet, and a vegetarian diet. Modeling diverse healthy dietary patterns ensures that the findings are applicable to a variety of dietary selections, making the results more relevant and adaptable for different population subgroups, and supporting broader dietary recommendations. A novel approach, the FNI, was used to assess the nutrient quality of eight underconsumed nutrients among adolescents in the selected menus. Moreover, the substitution was carried out based on an equal quantity (grams) of the replacement protein source food while holding energy constant, so the impact of specific food substitutions on overall dietary nutrient composition could be evaluated without confounding factors related to energy levels [65].

This study is also not without limitations. The modeling analysis is theoretical, the results are not actual intakes, and adolescents’ dietary intake when consuming such diets is unknown, as is the result of an intervention using the menus. However, the advantage of using specified substitution analysis in nutritional epidemiology has been previously demonstrated in studies of macronutrient intake and disease risk [66]. Another limitation concerns the restricted number of days of exemplary menus included in the price estimation, so future evaluations of price should include additional days of exemplary menus with egg substitution, as diet costs are considered a crucial driver of food purchasing decisions among low-income populations [62]. Additionally, the exclusion of spice costs in the HVEG menu and the use of similar food items when the exact food item was not available in the database may also limit the accuracy of the nutrient and price estimates. In addition, the energy levels of the exemplary menus do not fully align with the range of energy requirements for adolescent males, which may limit their applicability for adolescents with higher caloric needs. For these adolescents, the nutrient estimates may be lower than the levels that could be achieved using appropriate and strategic portion scaling or nutrient-dense additions to fulfill caloric needs. Consequently, while the evaluation of the modeled menus provides valuable insights into nutrient quality, these results should be interpreted with caution when generalizing to male adolescents with elevated energy demands.

## 5. Conclusions

In conclusion, substituting one egg for another protein-rich food increased the nutrient quality scores for choline in the HUSS, DASH, Harvard, and HVEG exemplary menus. The results of the current analysis also provide evidence that substituting one egg for another protein source food daily could play a role in alleviating choline inadequacy among U.S. adolescents. Nutrient quality for vitamin D modestly improved in the HUSS, DASH and HVEG menus. There was no significant difference in the average daily menu price with or without substituting one egg, so improvements in the choline and vitamin D profiles showed no price increases. Daily egg consumption has the potential to improve choline and vitamin D, two nutrients with notably inadequate intake among U.S. adolescents.

## Figures and Tables

**Table 1:** Total nutrients per day and comparison of daily mean nutrients across all menu days for each exemplary menu, as given and with the substitution of one egg, for the similar amount of another protein source food ^a^.

Day	Menu ^bc^	Choline ^d^	Potassium ^d^	Calcium ^e^	Folate ^e^	Magnesium ^d^	Zinc ^e^	Vitamin D ^e^	Vitamin C ^e^
				**HUSS** ^f^					
1	Menu as given	512.8	3485.1	1339.0	368.6	340.2	11.9	32.3	189.4
	Egg substitution	614.3	3447.8	1355.0	385.8	338.3	11.1	33.1	189.4
2	Menu as given	255.9	3875.7	1445.0	543.3	471.4	11.6	4.6	139.8
	Egg substitution	364.9	3858.2	1454.0	560.8	468.4	11.1	5.5	139.8
3	Menu as given	338.0	5003.1	1336.4	454.8	446.8	10.8	9.3	87.0
	Egg substitution	434.1	4961.1	1352.5	472.6	440.6	10.8	10.2	87.0
4	Menu as given	434.5	4141.1	1480.2	596.7	396.6	11.2	6.9	166.8
	Egg substitution	558.2	4099.3	1494.9	591.0	391.5	11.4	7.9	164.6
5	Menu as given	409.5	4317.4	1272.8	245.7	384.8	18.4	6.1	201.4
	Egg substitution	519.3	4315.7	1288.5	263.4	383.8	18.3	7.0	201.4
6	Menu as given	361.6	4582.3	1599.9	414.3	366.7	11.2	7.4	136.8
	Egg substitution	483.1	4411.8	1587.0	417.0	357.6	11.3	8.3	131.9
7	Menu as given	393.7	4800.3	1527.8	405.5	574.2	12.0	6.1	232.4
	Egg substitution	503.4	4600.3	1519.8	415.1	564.4	12.2	7.0	224.2
Daily mean menu as given		386.6	4315.0	1428.7	432.7	425.8	12.5	10.3	164.8
Daily mean egg substitution		496.7	4242.0	1436.0	443.7	420.7	12.3	11.3	162.6
				**DASH** ^g^					
1	Menu as given	408.9	4543.3	1322.1	397.3	559.4	15.3	9.1	143.0
	Egg substitution	512.9	4514.0	1338.0	413.8	555.5	15.1	10.0	142.8
2	Menu as given	311.3	4214.1	1483.5	510.2	534.4	11.4	5.7	98.1
	Egg substitution	407.4	4172.1	1499.6	528.0	528.1	11.3	6.6	98.1
3	Menu as given	383.9	4670.1	1533.2	837.7	636.3	14.2	8.6	211.8
	Egg substitution	479.2	4561.8	1548.4	854.9	634.3	12.7	9.6	211.8
4	Menu as given	404.3	4376.3	1474.7	343.1	457.4	12.6	8.9	112.3
	Egg substitution	493.6	4296.7	1493.0	361.1	455.2	11.8	9.5	112.3
5	Menu as given	377.7	4078.1	1409.5	521.2	469.5	13.5	7.4	174.3
	Egg substitution	475.8	4034.4	1419.3	534.5	461.5	13.0	8.2	173.7
6	Menu as given	414.3	4319.2	1399.8	531.5	504.4	10.1	8.3	252.9
	Egg substitution	414.3	4319.2	1399.8	531.5	504.4	10.1	8.3	252.9
7	Menu as given	282.7	4464.1	1489.7	603.0	549.3	14.0	10.2	75.4
	Egg substitution	388.7	4377.6	1498.3	619.2	535.5	13.9	10.2	75.4
Daily mean menu as given		369.0	4380.7	1444.7	534.9	530.1	12.9	8.3	152.5
Daily mean egg substitution		453.1	4325.1	1456.6	549.0	524.9	12.6	8.9	152.4
				**Harvard** ^h^					
1	Menu as given	425.4	4698.0	674.9	620.1	435.9	10.2	2.0	277.3
	Egg substitution	521.5	4656.1	691.0	637.9	429.6	10.2	2.9	277.3
2	Menu as given	297.3	3053.4	707.2	656.7	487.9	9.5	36.2	205.5
	Egg substitution	385.6	2915.9	721.0	665.6	477.5	9.8	28.4	196.0
3	Menu as given	388.7	3716.4	535.6	576.9	426.4	9.6	1.1	91.4
	Egg substitution	503.5	3708.1	534.9	581.6	423.3	9.8	2.0	90.6
4	Menu as given	382.8	3798.5	765.1	528.3	431.1	8.4	2.2	353.0
	Egg substitution	480.7	3742.1	751.1	521.5	413.1	8.2	3.0	351.1
5	Menu as given	364.7	3151.3	478.0	562.2	487.7	8.0	17.1	232.1
	Egg substitution	477.0	2962.6	464.1	536.9	468.4	8.0	18.1	220.4
6	Menu as given	511.7	4134.1	671.8	471.1	538.5	11.8	7.5	218.3
	Egg substitution	629.7	4088.3	665.8	483.2	527.7	11.6	8.5	215.3
7	Menu as given	604.9	3979.7	578.4	577.6	532.1	10.2	2.4	221.3
	Egg substitution	725.6	3937.5	585.2	581.9	520.9	10.4	3.3	217.8
Daily mean menu as given		425.1	3790.2	630.1	570.4	477.1	9.7	9.8	228.4
Daily mean egg substitution		531.9	3715.8	630.4	572.6	465.8	9.7	9.5	224.0
				**HVEG** ^i^					
1	Menu as given	606.3	4326.9	1739.7	988.6	549.1	15.8	7.3	123.2
	Egg substitution	703.7	4168.9	1726.5	957.4	523.8	15.6	8.3	123.2
2	Menu as given	350.0	4055.3	1688.0	730.2	596.0	13.1	7.4	79.1
	Egg substitution	462.0	3835.5	1665.8	709.8	569.5	12.9	8.3	79.1
3	Menu as given	539.3	4686.2	1450.2	900.1	409.1	13.0	6.4	79.3
	Egg substitution	649.4	4524.6	1461.0	813.0	392.3	12.7	7.3	78.4
4	Menu as given	372.2	4710.6	1367.0	990.1	629.9	19.8	6.8	197.2
	Egg substitution	485.4	4524.3	1367.0	1001.1	607.8	19.8	7.7	192.7
5	Menu as given	574.2	4183.1	1733.0	672.4	521.9	13.7	6.6	153.6
	Egg substitution	697.1	4155.9	1748.1	678.1	506.2	13.8	7.6	153.6
6	Menu as given	380.1	4297.0	1993.9	757.7	591.5	13.2	7.4	147.7
	Egg substitution	491.8	4290.7	1993.8	753.5	583.1	13.4	8.4	147.7
7	Menu as given	343.6	4794.6	1231.7	877.1	470.5	12.7	4.4	83.2
	Egg substitution	451.1	4660.5	1220.1	828.2	449.1	12.7	5.4	82.8
Daily mean menu as given		452.2	4436.2	1600.5	845.2	538.3	14.5	6.6	123.3
Daily mean egg substitution		562.7	4308.6	1597.5	820.2	518.8	14.4	7.6	122.5

^a^ One serving size of 44 g of an “egg, boiled (hard or soft)” from the National Data System for Research. ^b^ Menu as given = total nutrients for the exemplary menus as given. ^c^ Egg substitution = total nutrients for the modeling of the egg substitution where nutrients comprising the selected protein source from the menu were removed and the nutrients comprising one egg were included. ^d^ AI = Adequate Intake; choline AI = 400–550 mg, potassium AI = 2300–3000 mg, magnesium AI = 360–410 mg. ^e^ RDA = Recommended Dietary Allowances; calcium RDA = 1300 mg, folate RDA = 400 mcg, zinc RDA = 9–11 mg, vitamin D RDA = 15 mcg, vitamin C RDA = 65–75 mg. ^d,e^ AI and RDA ranges for adolescents 14–18 years are dependent on sex. ^f^ HUSS = Healthy U.S.-Style Dietary Pattern based on 2000 kcal/d. Standard deviation values for the daily mean for the exemplary menus as given and with the egg substitution, respectively, showed a range of values: choline ±80.5 mg to ±81.3 mg; potassium ±531.1 mg to ±496.4 mg; calcium ±117.6 mg to ±107.2 mg; folate ±115.3 mcg to ±110.8 mcg; magnesium ±79.5 mg to ±77.8 mg; zinc ±2.7 mg to ±2.7 mg; vitamin D ±9.8 mcg to ±9.7 mcg; and vitamin C ±48.3 mg to ±46.9 mg. ^g^ DASH = National Heart, Lung, and Blood Institute’s Dietary Approaches to Stop Hypertension (DASH) diet menu based on 2000 kcal/d. Standard deviation values for the daily mean for the exemplary menus as given and with the egg substitution, respectively, showed a range of values: choline ±51.6 to ±48.6; potassium ±200.1 mg to ±184.1 mg; calcium ±71.4 mg to ±73.1 mg; folate ±159.6 to ±159.7 mg; magnesium ±60.7 mg to ±61.0 mg; zinc ±1.8 mg to ±1.7 mg; vitamin D ±1.4 mcg to ±1.3 mcg; and vitamin C ±64.1 mg to ±64.1 mg. ^h^ Harvard = Harvard Medical School’s Healthy Eating Guide menu based on 2000 kcal/d. Standard deviation values for the daily mean for the exemplary menus as given and with the egg substitution, respectively, showed a range of values: choline ±102.4 mg to ±111.8 mg; potassium ±567.9 mg to ±616.4 mg; calcium ±102.2 mg to ±105.3 mg; folate ±60.1 mcg to ±64.5 mcg; magnesium ±47.3 mg to ±46.4 mg; zinc ±1.2 mg to ±1.3 mg; vitamin D ±13.0 mcg to ±10.1 mcg; and vitamin C ±79.0 mg to ±79.2 mg. ^i^ HVEG = Healthy U.S.-Style Vegetarian Dietary Pattern based on 2400 kcal/d. Standard deviation values for the daily mean for the exemplary menus as given and with the egg substitution, respectively, showed a range of values: choline ±115.5 mg to ±114.8 mg; potassium ±290.6 mg to ±284.0 mg; calcium ±262.7 mg to ±263.1 mg; folate ±126.7 mcg to ±121.4 mcg; magnesium ±77.7 mg to ±77.1 mg; zinc ±2.6 mg to ±2.6 mg; vitamin D ±1.1 mcg to ±1.1 mcg; and vitamin C ±45.6 mg to ±44.6 mg. No significant differences were found between the mean nutrients of the exemplary menus as given and the mean nutrients with the egg substitution based on a *t*-test, with *p* < 0.0001 to adjust for multiple comparisons using the Bonferroni method.

**Table 2:** Comparison of the daily means of the food nutrient index and component scores for exemplary menus as given and with the substitution of one egg for the similar amount of another protein source food ^a^.

FNI ^b^ Components	HUSS ^c^		DASH ^d^		Harvard ^e^		HVEG ^f^	
	Menu ^g^ Mean (n = 7)	Egg ^h^ Mean (n = 7)	Menu ^g^ Mean (n = 7)	Egg ^h^ Mean (n = 7)	Menu ^g^ Mean (n = 7)	Egg ^h^ Mean (n = 7)	Menu ^g^ Mean (n = 7)	Egg ^h^ Mean (n = 7)
Choline ^j^	83.4	95.1	79.6	91.1	88.6	98.3	91.1	100
Potassium ^j^	100	100	100	100	100	100	100	100
Calcium	100	100	100	100	48.4	48.4	100	100
Folate, DFE ^i^	100	100	100	100	100	100	100	100
Magnesium ^j^	100	100	100	100	100	100	100	100
Zinc	100	100	100	100	93.9	94.1	100	100
Vitamin D	69.2	75.2	55.4	59.4	65.1	63.0	44.1	50.5
Vitamin C	100	100	100	100	100	100	100	100
Total FNI score Mean ± SD	94.0 ± 2.3	96.3 ± 0.8	91.8 ± 2.2	93.8 ± 1.5	87.0 ± 3.0	88.0 ± 1.3	91.9 ± 1.5	93.8 ± 0.0
*p* value	0.04		0.07		0.44		0.01	

^a^ One serving size of 44 g of an “Egg, boiled (hard or soft)” from the National Data System for Research. ^b^ FNI = Food Nutrient Index ranging from 0 to 100. ^c^ HUSS = Healthy U.S.-Style Dietary Pattern based on 2000 kcal/d. ^d^ DASH = National Heart, Lung, and Blood Institute’s Dietary Approaches to Stop Hypertension (DASH) diet menu based on 2000 kcal/d. ^e^ Harvard = Harvard Medical School’s Healthy Eating Guide menu based on 2000 kcal/d. ^f^ HVEG = Healthy U.S.-Style Vegetarian Dietary Pattern based on 2400 kcal/d. ^g^ Menu = FNI daily means for the exemplary menus as given. ^h^ Egg = FNI daily means for the modeling egg substitution where nutrients comprising the selected protein source from the menu were removed and the nutrients comprising one egg were included; no significant differences were found between the mean nutrients of the exemplary menus and the mean nutrients with the addition of an egg based on *t*-test, *p* < 0.01 to adjust for multiple comparisons using Bonferroni method. ^i^ DFE, dietary folate equivalents. ^j^ Indicates an AI rather than an RDA. An AI is used when insufficient scientific evidence is available to establish the RDA.

**Table 3:** Comparison of the mean daily price ^a^ of the exemplary menus as given and with the substitution of one egg for the similar amount of another protein source food ^b^.

	HUSS ^c^ Menu Price (USD)		Harvard ^d^ Menu Price (USD)		DASH ^e^ Menu Price (USD)		HVEG ^f^ Menu Price (USD)	
	Menu as Given	Egg Substitution	Menu as Given	Egg Substitution	Menu as Given	Egg Substitution	Menu as Given	Egg Substitution
Day 1	7.6	7.4	6.0	5.9	5.7	5.6	8.0	7.7
Day 2	7.7	7.7	6.3	5.8	5.1	5.0	5.1	5.1
Day 3	9.9	9.8	5.4	5.4	6.7	6.1	6.5	6.6
Day 4	10.6	10.6	5.5	5.2	7.0	6.8	10.7	10.6
Day 5	10.3	10.1	7.9	7.8	5.5	5.5	5.5	5.5
Day 6	10.2	10.2	8.4	8.3	6.4	6.4	5.0	5.1
Day 7	11.0	10.8	5.1	5.0	6.1	5.9	6.7	6.8
Daily mean (SD)	9.6 (1.4)	9.5 (1.4)	6.4 (1.3)	6.2 (1.3)	6.1 (0.7)	5.9 (0.6)	6.8 (2.0)	6.8 (1.9)
*p* value	0.8		0.7		0.5		1.0	

^a^ Estimation of food prices using the Food Prices Database, 2003–2004 from the U.S. Department of Agriculture. ^b^ One serving size of 44 g of an “Egg, boiled (hard or soft)” from the National Data System for Research. ^c^ HUSS = Healthy U.S.-Style Dietary Pattern based on 2000 kcal/d. ^d^ Harvard = Harvard Medical School’s Healthy Eating Guide menu based on 2000 kcal/d. ^e^ DASH = National Heart, Lung, and Blood Institute’s Dietary Approaches to Stop Hypertension (DASH) diet menu based on 2000 kcal/d. ^f^ HVEG = Healthy U.S.-Style Vegetarian Dietary Pattern based on 2400 kcal/d. No significant differences were found between the mean nutrients of the exemplary menus and the mean nutrients with the addition of an egg based on *t*-test, with *p* < 0.01 to adjust for multiple comparisons using the Bonferroni method.

## Data Availability

The analytic code will be made available upon request due to privacy, or ethical reasons, pending application and approval.

## Supplementary Materials

The following supporting information can be downloaded at: https://www.mdpi.com/article/10.3390/nu17071129/s1, Table S1: Daily Protein Sources and Substitutions in the Healthy U.S.-Style Dietary Pattern (HUSS); Table S2: Daily Protein Sources and Substitutions in the National Heart, Lung, and Blood Institute’s Dietary Approaches to Stop Hypertension (DASH) Diet Menu; Table S3: Daily Protein Sources and Substitutions in the Harvard Medical School’s Heathy Eating Guide (Harvard Menu); Table S4: Daily Protein Sources and Substitutions in the Healthy and Vegetarian U.S.-Style (HVEG).

## Abbreviations

The following abbreviations are used in this manuscript:

DGA	Dietary Guidelines for Americans
USDA	U.S. Department of Agriculture
HUSS	Healthy U.S.-Style Dietary Pattern
DASH	National Heart, Lung, and Blood Institute’s Dietary Approaches to Stop Hypertension diet
HVEG	Healthy U.S.-Style Vegetarian Diet Pattern
FNI	Food Nutrient Index
