# Supplementary material for: Modeling the Substitution of One Egg Increased the Nutrient Quality of Choline and Vitamin D in Exemplary Menus
Source: Nutrients. 2025 Mar 24;17(7):1129. doi: 10.3390/nu17071129 (PMC11990234; doi:10.3390/nu17071129)
Supplement: Supplementary file 1 [file nutrients-17-01129-s001.zip › nutrients-3519301-supplementary.pdf]

Supplementary materials

Table S1. Daily protein sources and substitutions in the Healthy U.S.-Style Dietary Pattern (HUSS)

Day	Food Description	Protein source chosen for substitution (X)
1	Burrito, egg (with cheese), plain	
1	Salmon, cooked from fresh or frozen, unknown kind	
1	Roast beef, unknown cut, oven roast - dry heat, no visible fat eaten	X
2	Turkey, ground turkey, unknown % fat	X
3	Chicken, breast, skin removed before cooking	X
3	Tuna, canned, light, water pack, regular, drained - not rinsed	
4	Spaghetti - main dish (noodles and sauce), with tomato sauce, with meat, from recipe	X
4	Eggs, boiled (hard or soft)	
5	Steak - beef, unknown cut, no visible fat eaten	
5	Lunchmeats and sausages, chicken, chicken - deli style	X
6	Potato, baked, topped, with chili, skin eaten	X
7	Chowder, clam - Manhattan (tomato base), prepared from recipe	X

Table S2. Daily protein sources and substitutions in the National Heart, Lung, and Blood Institute's Dietary Approaches to Stop Hypertension (DASH) diet menu

Day	Food Description	Protein source chosen for substitution (X)
1	Chicken Salad (CHIX)	X
1	Steak - beef, unknown cut, no visible fat eaten	
2	Chicken, breast, skin removed before cooking	X
3	Steak - beef, sirloin, no visible fat eaten	X
4	Ham, regular cured, boneless, low sodium, no visible fat eaten	X
4	Chicken/Spanish rice (Chix rice)	
5	Tuna (Tuna)	
5	Turkey meatloaf/balls (Turkey)	X
6	Turkey, breast, unprocessed, skin removed before eating	X
6	Spicy baked fish (Spicy fish)	
7	Tuna, canned, light, water pack, regular, drained - not rinsed	X

Table S3. Daily protein sources and substitutions in the Harvard Medical School's Heathy Eating Guide (Harvard Menu)

Day	Food Description	Protein source chosen for substitution (X)
1	Chicken, breast, skin removed before cooking	X
2	Grilled Salmon wPapaya Mint Salsa (Grilled Salm)	X
3	Pork Tenderloin w Pistachio Gremolata Crust (Pork Tenderl)	X
3	Sandwich, chicken fillet on a bun, with grilled chicken, with lettuce and tomato	
3	Salad, lettuce, tossed, without dressing, with tomatoes and/or carrots, without avocado, cheese or egg	
4	Eggs, fried, whole egg	
4	Onion Crusted Tofu (Onion Cruste)	X
5	Chipotle, Chipolte Chicken Chili (ChickChili)	X
5	Fish and seafood, sea bass, unknown kind	
6	BWT Wrap (BWT Wrap)	X
6	Spicy Shrimp and Peanut noodle salad (Spicy Shrimp)	
6	Eggs, boiled (hard or soft)	
7	California Chicken Salad (CA CHXSal)	
7	Chipotle, Chipolte Chicken Chili (ChickChili)	X
7	Omelet, plain	

Table S4. Daily protein sources and substitutions in the Healthy and Vegetarian U.S.-Style (HVEG)

Day	Food Description	Protein source chosen for substitution (X)
1	Eggs, whole, raw	
1	Lentils, cooked from dried	
1	Tofu (soybean curd), silken, not cooked, regular	X
1	Green peas, cooked from frozen	
1	Milk, 1% fat or lowfat	
2	Milk, 1% fat or lowfat	
2	Peanut butter, regular, with salt	
2	Green peas, cooked from frozen	
2	Beans, great northern, canned - drained, regular	X
2	Peanuts, roasted, oil roasted, lightly salted	
2	Milk, 1% fat or lowfat	
3	Yogurt, Chobani Fruit On the Bottom Non-Fat Greek Yogurt - all flavors	
3	Cheddar cheese, natural	
3	Lentils, cooked from dried	X
3	Lima beans, cooked from frozen	
3	Eggs, boiled (hard or soft)	
3	Milk, 1% fat or lowfat	
4	Milk, 1% fat or lowfat	
4	Refried beans, canned, regular	X
4	Chickpeas, canned - drained, regular	
4	Peanut butter, regular, with salt	
5	Swiss cheese, natural	
5	Provolone cheese, natural	
5	Beans, black, canned - drained, regular	
5	Peanuts, roasted, oil roasted, salted	X
5	Milk, 1% fat or lowfat	
5	Eggs, whole, raw	
5	Greek yogurt, plain, nonfat (<1% fat)	
6	Milk, 1% fat or lowfat	
6	Green peas, cooked from frozen	
6	Chickpeas, canned - drained, regular	X
6	Beans, great northern, canned - drained, regular	
6	Cottage cheese, lowfat (2% fat)	
6	Milk, 1% fat or lowfat	
7	Greek yogurt, fruit flavors, regular, nonfat (<1% fat)	
7	Beans, black, canned - drained, regular	X
7	Milk, 1% fat or lowfat	
